# Caregiver worry and injury hazards in the daily lives of Ugandan children

**DOI:** 10.5249/jivr.v13i1.1515

**Published:** 2021-01

**Authors:** Lindsay M. Stager, Marissa Swanson, Emma Hahn, David C. Schwebel

**Affiliations:** ^*a*^ Department of Psychology, University of Alabama at Birmingham, Birmingham, AL, USA.; ^*b*^ Department of Psychology, Emory University, Atlanta, GA, USA.

**Keywords:** Wounds and Injuries, Safety, Child, Uganda

## Abstract

**Background:**

Over 95% of unintentional injury-related childhood deaths globally occur in low- and middle-income countries, such as Uganda. Risks for injury in settings like rural Uganda are vastly understudied despite differing patterns of child injury risk. The present study investigated the prevalence and type of hazards in children’s environments in rural Uganda, as well as the relationship between hazard exposure and parent attitudes and perceptions regarding unintentional injury.

**Methods:**

Our sample included 152 primary caregivers in Eastern Rural Uganda who had children in either 1st or 6th grade. All parents/guardians completed caregiver surveys following verbal instructions. Surveys assessed demographic information, child hazard exposure, and parent beliefs regarding child injury.

**Results:**

Almost all parents (98.5%) reported daily exposure for their children to at least one of the hazards assessed. Caregiver's perceived likelihood of child injury was positively related to hazard exposure (r = .21, p less than .05). This relationship remained significant when controlling for family demographics, child grade level, and child injury history (F (7, 126) = 2.25, p less than .05).

**Conclusions:**

Our results suggest that Ugandan parents are aware of the risks of children’s exposure to hazards, but may lack the tools to address it. Development of injury prevention interventions focusing on behavioral change techniques may help reduce childhood injury and injury-related deaths in Uganda.

## Introduction

Childhood injury presents substantial risk to children in low- and middle- income countries (LMICs) as over 95% of all global unintentional injury-related childhood deaths occur in LMICs.^1^ In Uganda, a child under the age of 10 is estimated to have over 1.5 times the risk of an injury-related death compared to a child the same age in the United States.^2^ Overall, the Demographic Health Survey 2016 reported that 899 Ugandan children under the age of 15 experienced a serious unintentional injury in 2016. This number included 65 children who died from their injuries.^3^ Correspondingly, data from the Global Burden of Diseases 2019 study estimated that injuries accounted for approximately 16.36% of deaths in children ages 5-14 years old in Uganda in 2019.^4^

Child injury rates differ greatly between rural and urban areas in most global locations, as rural areas tend to have differing patterns of child injury risk and limited access to quality medical resources.^5,6^ A highly systematic survey of residents in one rural and one urban setting in Uganda in 1997 and 1998, for example, suggests higher rates of drowning and falls in rural areas, as compared to more urban areas where children have higher rates of traffic-related injuries and burns.^6^ The authors concluded these differences likely relate to the unique environments created by rural and urban settings in Uganda. For example, higher rates of drowning in rural areas may relate to greater exposure to unprotected bodies of water, while traffic-related injuries in urban areas stem from increased population density and heavier road traffic.^6^

However, these data are over two decades old, and just a few studies have considered hazard prevalence and childhood injury in rural Uganda since that time. One more recent study, from 2008 at an urban hospital in Kampala, found child injuries were most likely to occur in the home (over 50% of treated injuries) or on the road (about 30%) and during play. Some developmental trends emerged concerning types of injuries, with younger children experiencing burns most often, and falls and traffic-related injuries increasing in frequency as children grew older.^7^ A 2011 study supports these results, suggesting falls, burns, and cuts were the most frequent injuries for children in both rural and urban areas of Uganda.^8^ However, conflicting data from a rural mission hospital in Kuluva highlighted falls, traffic injuries, and snakebites as the top causes of child injury.^9^

Because of the limited data on child injuries in Uganda, and especially given the vast differences in the environmental risks children encounter in rural versus urban Uganda, further exploration of hazard exposure is warranted to guide empirically-supported intervention development.

Also unclear are the factors that may lead to child injuries in rural Uganda. Global research conceptualizing injury risk factors stresses the influence of multiple factors. A qualitative study of caregivers of children in South Africa who had sustained burn injuries, for example, cites the coinciding risk emerging from the child’s activities, the caregiver’s activities, and the environment the child is engaging within.^7^ One key factor cited broadly by interventionists is the caregivers’ perceptions of child injury risk, as this may alter hazard exposure patterns. Health behavior change models such as the Health Belief Model provide a framework through which these relationships may be understood.^10^ According to the Health Belief Model, caregivers may perceive greater vulnerability to injury in their children due to 1) personally held beliefs that injuries are possible or even likely, 2) elevated levels of worry focused on injuries, and/or 3) experience of actual injuries in the recent past. As these factors increase a child’s perceived vulnerability in the eyes of their parent, the Health Belief Model suggests that the same factors may also motivate parents to make behavioral changes aimed at improving their child’s safety, assuming perceived and actual self-efficacy to make those changes.^11^ Thus, elevated parental worry regarding child injury or perceived likelihood of child injury may relate to lower levels of hazard exposure if parents believe hazard exposure can be reduced. The same would be true about recent injury experiences and subsequent reductions in hazard exposure. 

The present study investigated the prevalence and types of hazards in the daily living environments of children in rural Uganda, as well as the relations between hazard exposure and three different factors that may influence parents’ perceived vulnerability to child injury: caregiver perception of the likelihood of child injury, caregiver worry about injuries, and caregiver reports of major and minor child injuries in the past year. We considered these factors among a sample of 152 families, focusing on hazards found within children’s homes (i.e., paraffin, knives, etc.) and the surrounding area (i.e., bodies of water, large animals, and open pits). We predicted that overall rates of hazard exposure would be high and that higher parent levels of worry, greater perceived likelihood of injury, and a history of past year minor or major childhood injury would relate to lower child hazard exposure. Within our models, we examined the possible influence of parent gender, child age, parent age, parent education, and household socioeconomic status as potential covariates.

## Methods 

**Participants**

Participants were recruited as part of a larger intervention study that sought to assess the efficacy of a manualized, classroom-based program to promote safety behaviors in Ugandan students.^12^ This program focused on teaching personal safety skills to first grade students and teaching child supervision skills to sixth grade students. All data presented herein were collected at baseline, prior to any intervention.

**Table 1:** Demographic data.

Caregiver Demographics (N=152)		%
Gender	Male	39.5%
	Female	55.4%
	No Response	5.3%
Home Construction	Mud, Iron, or Timber	80.9%
	Brick	15.9%
	No Response	3.3%
Education	Less than a Primary Education	27.2%
	Primary Education	19.6%
	More than a Primary Education	46.2%
	No Response	7%
Age (years)	<18	1.3%
	18-24	6.4%
	25-29	7.6%
	30-39	29.9%
	40-49	35.0%
	50-59	12.7%
	60-69	2.5%
	No Response	4.6%
**Child Demographics (N=152)**		
Grade Level	1st	41.4%
	6th	49.0%
	No Response	9.8%

The intervention was administered at local primary schools in rural Eastern Uganda. Thus, all participants were recruited through local schools. In total, 152 primary caregivers of first (ages 6-9) and sixth (ages 11-15) graders participated. The age groups were selected based on the hypotheses for the larger study, which concerned child safety among younger children (Grade 1) and child supervision among older ones (Grade 6). Verbal fluency in either English or Lugisu – the native language of the area – was required to participate, but literacy was not required. No eligible participants were excluded for language ability or for any other reason. Caregiver and child demographics appear in Table 1.

**Procedure**

Participants consented to be involved in the study during a parents’ meeting at the school. Schools facilitated the meeting and invited parents to attend, but the research team provided all information about the study and coordinated informed consent processes. All parents provided informed consent and then completed caregiver surveys following verbal instructions. Specifically, surveys were read to caregivers in their preferred language (either English or Lugisu) by trained local research assistants and responses were indicated by marking verbally-explained symbols on a response sheet. Symbols included visual representations of response options, such as pictures of houses built from different materials (mud/sticks, polythene sheets, timber, stone/bricks) or answer circles of increasing size depicting increased levels of worry or perceived likelihood regarding injury. If caregivers had multiple children in first and/or sixth grade, data for one child were randomly selected for inclusion in the present analysis to avoid shared variance in statistical analyses. Participating caregivers received a small household item, such as a bar of soap, as compensation. The protocol was reviewed and approved by the Institutional Review Board at the University of Alabama at Birmingham and the Mildmay Uganda Research Ethics Committee (REC), a national REC accredited by the Ugandan National Council for Science and Technology.

**Measures**

Participants completed a novel self-report questionnaire adapted to fit the culture of rural Eastern Uganda. Questionnaire items addressed four domains:

Basic Demographics: Participants reported their gender, age, and child’s grade level. Caregivers also reported their total years of formal education using a 7-point scale appropriate for the educational system in Uganda (0=none, 1=some primary school, 2=primary school, 3=O’level or lower level secondary school, 4=A’level or upper level secondary school, 5=university, 6=technical). Education was subsequently grouped into three categories: 1) less than a primary education, 2) primary education, 3) more than a primary education. This grouping allowed for analysis of education as an ordinal variable with sufficiently large and relatively equal cell sizes.

Home Construction: Home construction was used to approximate participant socioeconomic status (SES).^13^

Home construction was assessed using the following question: “What type of material are the walls in your main house made from?” Having brick walls on their home identified participants as having higher SES, while families who had walls made from mud, iron, or timber were considered more typical of lower SES. 

Hazard Exposure: The questionnaire assessed child exposure to regionally common hazards in and around the home. Parents indicated whether or not children currently had access to knives, axes, cooking areas, paraffin (kerosene), large animals (i.e., cows, goats, pigs), water sources for fetching to use in the home, water sources such as bodies of water where laundry is washed, glass, medicine, open pits, electrical wires, chainsaws, petrol (gasoline), alcohol, and dogs at home (Table 2). Items included yes/no questions such as, “Does your child have access to petrol in your home?” A total hazard exposure variable was computed by summing the total exposures for all of these variables (no=0, yes=1; range: 0-15). The measure was designed to represent children’s exposure to common hazards but not to be comprehensive in measuring exposure to all potential injury hazards in the child’s environment.

**Table 2:** Exposure of First and Sixth Graders to Common Hazards (N=152).

Hazards	1st Grade n (%)	6th Grade n (%)	χ2	p
Knives	63 (89%)	69 (90%)	.03	1.00
Axe	62 (87%)	71 (92%)	.97	.48
Cooking Area	62 (86%)	70 (91%)	.85	.51
Paraffin	50 (69%)	53 (70%)	.00	1.00
Large Animals	48 (67%)	62 (80%)	3.15	.11
Water – Fetching	47 (66%)	41 (54%)	2.29	.18
Glass	47 (66%)	50 (66%)	.00	1.00
Water – Laundry	43 (62%)	53 (69%)	.69	.51
Medicine	37 (52%)	40 (52%)	.00	1.00
Open Pits	23 (33%)	38 (49%)	4.10	.06
Electrical Wires	15 (22%)	18 (24%)	.08	.94
Chainsaw	8 (11%)	14 (18%)	1.24	.38
Petrol	6 (9%)	7 (10%)	.05	1.00
Alcohol	4 (6%)	6 (8%)	.25	.86
Dog at Home	3 (4%)	9 (12%)	2.68	.18

Note: p-values reported with Bonferroni correction. No significant differences in exposure rates observed between groups after Bonferroni correction for multiple comparisons.

Caregiver Worry: Caregivers reported worry about child injury with 3 items: “how much do you worry about your children… 1) getting a minor injury that does not need to be treated at a health center? 2) getting a major injury that needs to be treated at a health center? and 3) dying from an injury?” Each item was scored using a 4-point Likert scale ranging from 0 (not at all) to 3 (a lot). Overall scores for caregiver worry were computed by averaging the scores for each of the three individual items (range: 0-3).

Caregiver Perceived Likelihood of Injury: Caregivers reported perceived likelihood of child injury with 3 items: “How likely do you think it is for your children to… 1) get a minor injury? 2) get a major injury? 3) to die from an injury?” Each item was scored using a 4-point Likert scale ranging from 0 (no chance) to 3 (large chance). Overall scores for caregiver perceived likelihood of an injury were computed by averaging the scores for each of the three individual items (range: 0-3).

Child Injury History: Caregivers reported child injury history using 2 items: whether the child had experienced at least… 1) one major (i.e., needed treatment at a health center) injury in the past year, 2) one minor (i.e., did not need treatment at a health center) injury in the past year. Each item was answered and scored as either no (0) or yes (1). Overall scores for injury history were computed by averaging the scores for the two individual items (range: 0-1).

**Data analyses**

Analyses proceeded in three steps. First, prevalence rates for hazard exposure in each child age group were assessed and chi-square tests were computed to compare exposure rates for the 15 different hazards between age groups, with a Bonferroni correction applied for multiple comparisons between the same age groups. Second, correlations were used to assess unadjusted relations between family demographics (caregiver education, age, gender, and home construction), child injury history, caregiver worry about injuries, caregiver perceived likelihood of injuries, and child hazard exposure. Third, two linear regressions predicted child hazard exposure using measures of both caregiver worry about injuries and caregiver perceived likelihood of injuries along with family demographics, child grade level, and child injury history. All statistical analyses were conducted using SPSS software version 27.0. A cutoff of p <.05 was used to establish statistical significance.

## Results

Hazard exposure rates for first and sixth graders appear in Table 2. As predicted, overall hazard exposure was high among both groups, with over 50% of all parents reporting child exposure to the following hazards: knives, axes, cooking areas, paraffin, large animals, bodies of water, glass, and medicine. Almost all parents (98.5%) reported daily exposure for their children to at least one of the hazards assessed. No significant differences in hazard exposure were found between the grade levels. 

Table 3 shows descriptive data for, and correlation values between, demographic variables; caregivers’ perceived likelihood and worry regarding child injury; child injury history; and total hazard exposure. Demographics were generally not related to any of the primary variables of interest (caregiver worry, perceived likelihood of injury, child injury history or total hazard exposure), with one exception: parent education correlated significantly with child injury history (r = .20, p <.05).

**Table 3:** Correlations between demographics, injury history, parental injury variables, and hazards (N=152).

Variables	M	SD	1	2	3	4	5	6	7
1. Parent Gender	—	—	—						
2. Parent Education	2.20	.87	-.05	—					
3. Parent Age	—	—	-.16	-.19*	—				
4. Home Construction	.16	.37	.20*	.13	-.09	—			
5. Caregiver Worry	2.03	.66	.09	.15	-.13	-.04	—		
6. Caregiver Perceived Likelihood	1.75	.56	.16	.00	-.11	.04	.55**	—	
7. Injury History	.38	.34	-.08	.20*	-.12	-.07	-.01	-.06	—
8. Total Hazard Exposure	7.43	2.65	-.07	-.03	.11	-.09	.09	.21*	.02

Note: M (mean) and SD (standard deviation) omitted for categorical variables. * = p<.05, ** = p<01.

Caregiver’s perceived likelihood of a child injury was significantly related to total hazard exposure. However, the effect of this relationship fell in the opposite direction of our hypothesis. Greater levels of child hazard exposure were related to higher levels of overall caregiver perceived likelihood of injury (r = .21, p <.05). This relationship remained significant when controlling for family demographics, child grade level, and child injury history in a multiple regression model (F (7, 126) = 2.25, p <.05; Table 4). 

**Table 4:** Summary of linear regression models predicting child hazard exposure.

	B	SE B	β	p
**Caregiver Worry (N=134)**				
Caregiver Worry	.55	.34	.14	.11
Caregiver Education	-.06	.28	-.02	.84
Caregiver Age	.35	.22	.16	.11
Caregiver Gender	-.24	.47	-.05	.61
Home Construction	-.52	.62	-.08	.40
Child Grade	.07	.09	.07	.45
Child Injury History	.56	.68	.07	.41
**Caregiver Perceived Likelihood (N=134)**				
Caregiver Perceived Likelihood	1.21	.40	.26**	<.01
Caregiver Education	-.02	.27	-.01	.95
Caregiver Age	.37	.21	.16	.09
Caregiver Gender	-.37	.46	-.07	.42
Home Construction	-.58	.61	-.08	.34
Child Grade	.06	.09	.05	.55
Child Injury History	.71	.66	.09	.29

Note: *=p.05m, **=p<.01.

No significant relations were observed between caregiver worry regarding child injury and child hazard exposure (r = .30, p=.09) in the unadjusted correlations or in an adjusted regression model controlling for family demographics, child grade level, and child injury history (F(7, 126) = 1.30, p = .25). 

## Discussion

The rural Ugandan children in this study faced exposure to high levels of household hazards. Based on underlying theory from the Health Belief Model,^10^ we hypothesized that higher levels of caregiver worry about injury, higher caregiver perceived likelihood of child injury, and a positive history of recent injuries among their children would be related to lower levels of hazard exposure. Contrary to our hypotheses, we found that caregivers who expressed greater perceived greater likelihood of injury to their children had children with higher exposure to hazards.

In some ways our results, though contrary to the hypothesis, are logical. The Health Belief Model was developed and may fit best in an environment where parents are resourced to create health behavior change. Among our sample, parents may lack adequate resources to instigate health-related change to improve their children’s safety. In theoretical terms, parents need self-efficacy – both perceived and actual – to instigate health-related behavior change. Thus, while Ugandan parents may be aware of the potential harm from hazards in their children’s environments, they may require support and resources to address those concerns. As such, those parents whose children are exposed to more hazards accurately perceive injuries to be somewhat more likely than those parents whose children are exposed to fewer hazards.

We found that there was similar exposure to hazards across our samples of 1st and 6th graders. Overall, younger children in Uganda experience higher rates of home injuries than older children^8^ and have greater rates of injury mortality.^14^ These differences may be driven by developmental differences rather than variations in hazard exposure. Thus, hazard reduction in the living environments of younger children may be especially critical to reducing child injury risk.

Our findings highlight the need for empirically supported interventions to improve child safety in rural Uganda. Current research indicates a lack of safe hazard storage and inadequate child supervision, highlighting key areas for intervention.^8,12^ Additionally, while the national school curriculum in Uganda mandates training in safety, only about 5% of teachers in rural Uganda report receiving explicit instruction in how to teach safety lessons to children.^15^ If teachers could offer an empirically-supported training program with active learning techniques to promote safer child behavior, this may allow children to be safer even when they engage in somewhat hazardous environments. Recent research demonstrates the effectiveness of multiple interventions for child and adolescent risk reduction, including interventions focused on increased self-efficacy of at risk populations, hazard related education, and the role of peer influence.^16-18^ However, it is important to note that additional interventions outside of the school, such as distribution of safety-related devices (e.g. safe storage containers) are recommended to supplement school-based efforts to yield the most effective child safety outcomes.^19^

Our study is unique in considering hazard prevalence and childhood injury risk in rural Uganda. However, there are limitations which should be considered when interpreting the findings. First, data for this study were cross-sectional, and therefore we are unable to address possible causality in the results. Data were also based entirely on self-report. While this allowed us to begin understanding hazard exposure in rural Uganda, future research might implement observational strategies to consider more closely the precise areas of behavior change that could be valuable to guide future intervention efforts. Third, we relied on parents’ retrospective recall for variables such as child injuries over the past year. Although there is evidence that parents have reasonable memory of events like major injuries over the past year,^10^ this reliance may impact validity of our data, especially for more minor injury events. ^21^ Fourth, it is hard to generalize our results to other LMIC populations, as our population was specific to one district in eastern rural Uganda. We also included only children who were attending school, a factor that may limit generalizability to all children living in the region. Finally, our focus on only two age groups may limit our understanding of hazard exposure across all of child and adolescent development.

Overall, this study expands on our knowledge concerning hazard exposure in LMICs like Uganda, a critical step toward developing effective intervention programs. The results suggest that while parents may be aware of the risk presented to their children by increased hazard exposure, children in rural Uganda face high amounts of environmental hazards in their daily living environments regardless. Development of multifaceted empirically supported injury prevention interventions may help reduce unintentional childhood injury and death in Uganda and other LMICs.

**Acknowledgements**

The authors thank our participants, staff, and school collaborators for their support and efforts on behalf of this project.
